# Malignant hyperthermia and surnames: ethical dilemmas and diagnostic pitfalls

**DOI:** 10.1016/j.bjane.2025.844622

**Published:** 2025-04-19

**Authors:** Helga Cristina Almeida da Silva, Maria Anita Costa Spindola, José Luiz Gomes do Amaral

**Affiliations:** a Universidade Federal de São Paulo, Departamento de Anestesiologia, Dor e Medicina Intensiva, São Paulo, SP, Brazil; b Universidade Federal de Santa Catarina, Hospital Universitário Polydoro Ernani de São Thiago, Florianópolis, SC, Brazil

Dear Editor,

As members of Malignant Hyperthermia (MH) diagnostic centers in Brazil, we received several requests from healthcare professionals for a list of surnames of patients who experienced MH crises or tested positive for MH. Likewise, we received patients with no personal or family history of MH referred because their surnames were linked, in the past or in other locations, to someone suspected of MH. And recently a patient who had a family history of MH with death contacted us after he was discredited because his name was not on the list of people susceptible to MH circulating in the region. The accumulation of these facts led us to write this letter, in order to alert the anesthesiology community about the disadvantages of these practices.

MH is an autosomal dominant pharmacogenetic hypermetabolic syndrome triggered by halogenated anesthetics and/or succinylcholine in susceptible individuals. Although initially described in Caucasians, it occurs in all ethnic groups and still has a high mortality rate in Brazil.^1^ Population studies indicate that, although the frequency of MH crises varies between 1:10,000 in children and 1:50,000 in adults, the prevalence of people with mutations that cause MH may be as high as 0.46%.^2^ In this sense, the anesthesiologist must be prepared not only to diagnose and treat MH crises quickly but also to identify patients at risk, in order to perform anesthesia without triggering agents when necessary.

Attention to MH crises is one of the points listed in the Declaration of Helsinki on patient safety in anesthesiology, of which the SBA is a signatory.^3^ An article discussing this declaration emphasizes the need for protocols ensuring the immediate recognition of MH and availability of necessary drugs. Brazilian Federal Council of Medicine Resolution 2174/2017,^4^ which regulates the anesthetic procedure, expresses these MH guidelines in its text. It is worth noting that Brazil also has a telephone guidance service (Brazilian MH Hotline) and an informational website for MH (cedhima.unifesp.br).

The anesthesiologists who witness the MH crises contribute to the prevention of new MH crises, with the possibility of sequelae and death, when they provide the patients and their primary physician with written information about MH, genetic inheritance, and the need to inform relatives about this condition, in addition to referring the patient to investigation.^5^ MH centers, of course, keep records of cases and families, which can be useful in identifying other patients' connections with a new index case, and whether or not there is confirmation of the suspicion of MH in the family of each patient.^6^ In other words, based on a family history, look for any family information/biopsy about each new patient. The pre-anesthetic evaluation is the preferential moment for the professional to identify the personal and/or family history suggestive of MH and thus seek support from specialized centers and plan safe anesthesia for their patient.^5^ The most recent guidance protocols on the subject emphasize the importance of the pre-anesthetic evaluation.^5^^,^^6^

However, there is no scientific or ethical support in the opposite direction: there is no justification for the MH center to release a list of surnames. Several other reasons can also be cited: 1) Medical confidentiality; 2) General Data Protection Laws; 3) This creates a false sense of security, as some surnames may not appear on official documents, and family ties may be obscured by name changes through marriage or omission of maternal surnames. Therefore, a surname alone does not allow for reliable identification of family ties; 4) Possibility of more than one mutation occurring in the same family or of a de novo mutation (mutation absent in the parents but present only in the affected child).

In conclusion, the essential recommendations to prevent MH crises include thorough collection of personal and family history, written guidance for patients, referral to diagnostic centers, and avoiding exposure to triggering agents until susceptibility is ruled out.

## Funding

This study was performed with the support provided by the Coordination for the Improvement of Higher Education Personnel-Brazil (CAPES) ‒ Financing Code 001, and FAPESP (2021/06180-7).

## Conflicts of interest

The authors declare no conflicts of interest.
